# Follow, select and assemble method FSAM for VE and re assembly

**DOI:** 10.1016/j.mex.2020.100965

**Published:** 2020-06-19

**Authors:** Salvador Rojas-Murillo, Priyadarshini Pennathur

**Affiliations:** a Hofstra University, 1000 Hempstead Turnpike, Hempstead, New York 11549, United States; b The University of Iowa, Iowa City, IA 52242, United States

**Keywords:** Assembly, Eye-tracking, Training, Virtual vs real

## Abstract

We modified the methods by Ballard et al. [1]. They sought to study eye-hand coordination strategies and created both a real world and a virtual model copying task consisting of three different areas: model, source, and workspace. Participants followed a pickup and place exercise and used a mouse to control stimuli presented on a computer monitor in the virtual task. We also considered the method presented by Hayhoe et al. [2] and Aivar et al. [3] who designed a similar model copying task in a 3D virtual environment. Stimuli were displayed in a head mounted display and participants held a motion sensor to select and move virtual objects in 3D space. Moreover, Aivar et al. [3] also included extra assembly pieces and a variation in the position of the assembly pieces located in the resource area.

• It proposes an assembly task designed at a 1:1 scale for two environments, real and virtual environments.

• It introduces a reading sequence for the model that is being replicated and it also introduces distractor assembly blocks with similar colors and shapes as the required assembly blocks, and a change in the location for all assembly blocks in the resource area.

• It modifies the interaction for the VR environment by using hand gestures to select, move and position virtual assembly blocks. This was possible by incorporating a LEAP® motion controller which although it does not provide haptic feedback, it provides a virtual representation of the participant's hand. Our VE also includes visual and auditory feedback to guide depth perception and virtual control.

The software used for this research study is available at: http://virtualete.com/research/fsam.php

## Graphical abstract

**Figure:**
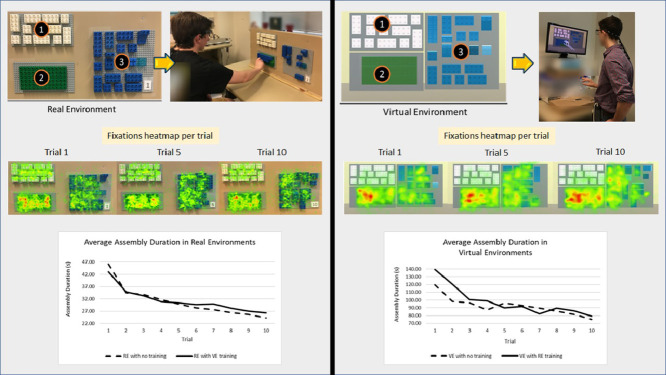

Specifications Table

**Table:** 

Subject Area	Engineering
More specific subject area	Human factors and ergonomics.
Name and reference of original method	[1] D. H. Ballard, M. M. Hayhoe, and J. B. Pelz, “Memory Representations in Natural Tasks,” J. Cogn. Neurosci., vol. 7, no. 1, pp. 66–80, Jan. 1995. The method involved a pick and place task to copy a pattern of colored blocks. In a virtual environment using a computer mouse to interact with the model The visual display had three areas, the model, source, and workspace. The model area contained the visual instruction that had to be followed; the source contained the assembly blocks and the workspace was where assembly blocks were assembled following the pattern of the visual instructions. [2] M. Hayhoe, P. Aivar, A. Shrivastavah, and R. Mruczek, “Visual short-term memory and motor planning,” in Progress in Brain Research, vol. 140, J. Hyona, D. P. Munoz, W. Heide, and R. Radach, Eds. Elsevier, 2002, pp. 349–363. The method involved the preparation of a sandwich in a real environment and the assembly of virtual toy using a VR head-up display and a glove to interact with the virtual environment. [3] M. P. Aivar, M. M. Hayhoe, C. L. Chizk, and R. E. B. Mruczek, “Spatial memory and saccadic targeting in a natural task,” J. Vis., vol. 5, no. 3, pp. 3–3, Mar. 2005, doi: 10.1167/5.3.3. The method involved the assembly of virtual toy using a VR head-up display and a glove to interact with the virtual environment with extra assembly parts.
Resource availability	Images are attached to this manuscript

*Method details Assembly task and assembly areas

We developed an assembly task that required participants to assemble virtual and real Lego Duplo® blocks.

## Participant selection and training

Participants’ participation was voluntary and received a gift card as compensation for their participation. To provide experimental consistency we recruited undergraduate and graduate male engineering students from the University of Iowa. Participants were screened for normal vision, physical capacity and eye-tracking capabilities. Normal vision was assessed for visual acuity and for color blindness using two different online tests. For visual acuity we used Essilor.com [1], and for color blindness we used EnChroma.com [2]. physical capacity was assessed by simple observation participants were able to stand, sit and move objects with their dominant hand without any physical impairments. Eye-tracking capabilities were assessed when calibrating the eye-tracker. Participants with very low contrast between the pupil and the iris were dismissed as the eye-tracker could not properly track eye movement.

Our methods were IRB approved. Prior to their performing the experiment participants read and signed the consent form. The consent form instructed participants on the experimental setting, how to perform the assembly process, how to read the visual instructions, how to differentiate between assembly blocks and distractors, how to manipulate the virtual environment, and invited participants to complete the assembly process as quickly as possible.

We split participants into two groups of 15 participants. The first group performed the assembly in the real environment first while the second group performed the virtual environment first. In addition, prior to performing the virtual assembly all participants had a training session where they were able to learn the hand gesture needed to pick, move and place virtual objects. The gesture is called a pinch gesture in which participants insert their virtual thumb into the block they want to select and move and then they join the tip of their index finger with the tip of their thumb. This gesture is easy to learn and maintain. Participants had to maintain this gesture to continue controlling the virtual block. In addition, the training session consisted of ten consecutive exercises which provided information about the visual and auditory feedback cues. Furthermore, participants could repeat the training exercise if the participant needed more practice. After receiving instructions and training, participants completed ten assembly cycles in both real and virtual environments. Participants were recommended to perform each cycle as quickly as possible.

## Experimental layout

**Fig. 1:**
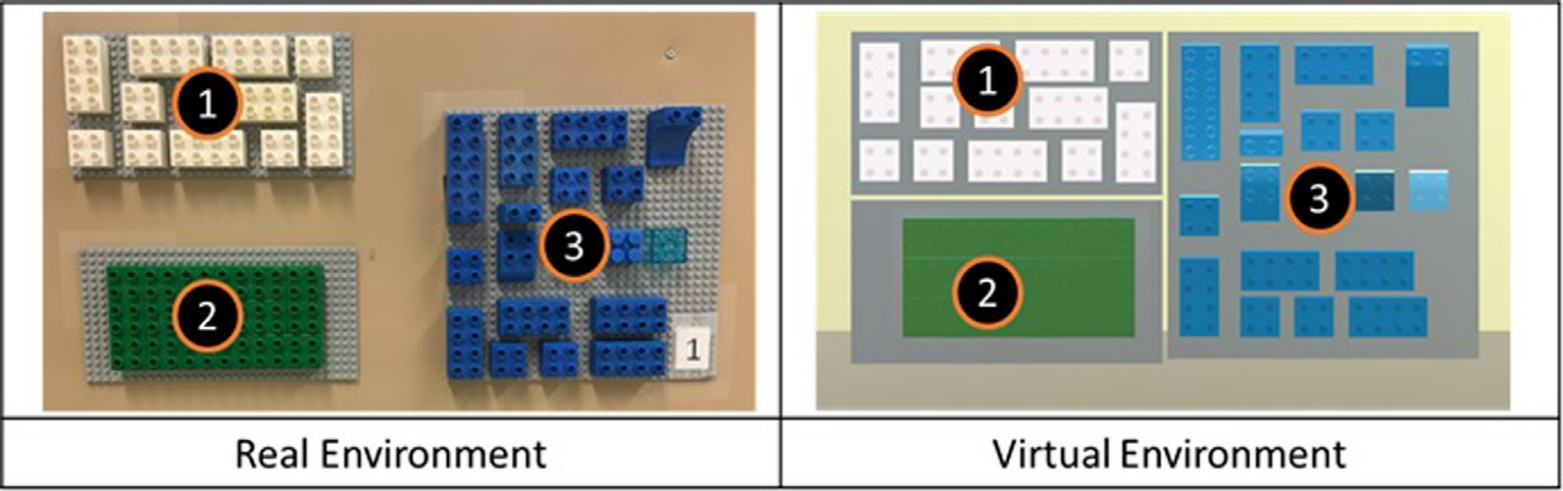
Real Environment and Virtual Environments Assembly Layout.

**Fig. 2:**
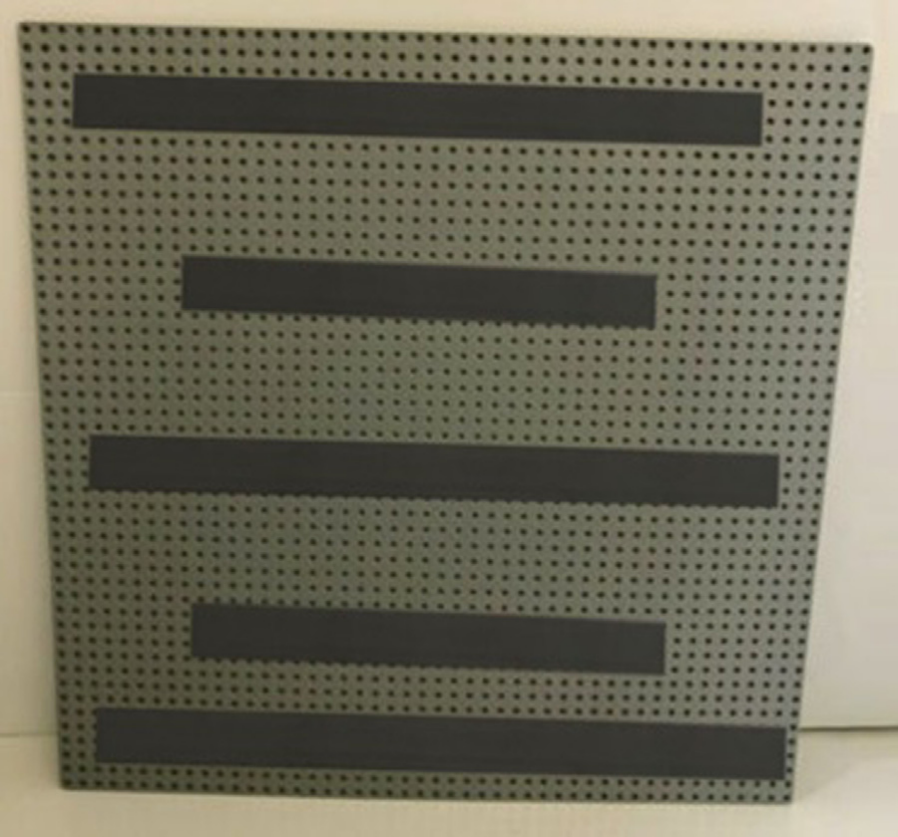
Back of a Duplo® panel with magnetic strips.

**Fig. 3:**
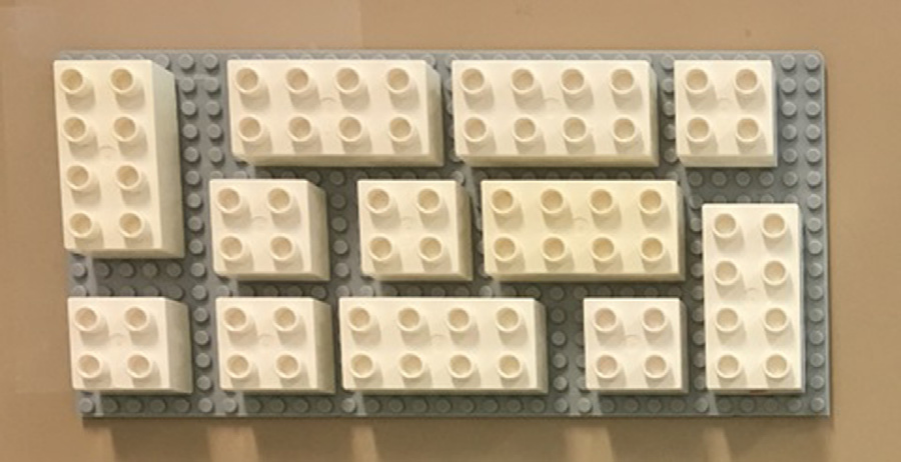
Visual Instruction pattern.

**Fig. 4:**
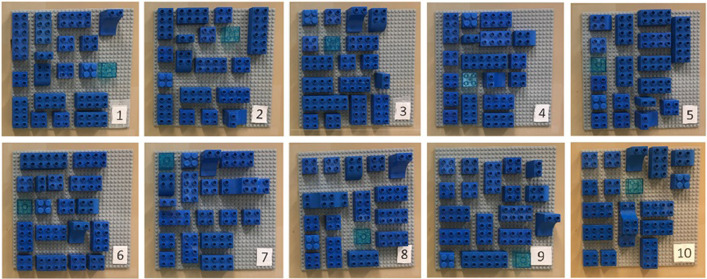
The ten resource areas.

We followed an assembly layout similar to the proposed by Ballard et al. Fig. 1, shows the three main areas of the assembly lay-out for the real and virtual environments. Area one, is the visual instruction pattern shown in Fig. 2, area two is the assembly area where blocks are assembled shown in Fig. 4, and the resource area with the assembly blocks and distractors is shown in Fig. 3. We used different colors to distinguish these layout areas.

The Lego Duplo® blocks were placed on a neutral grey color baseplate that had a different color than the metal panel where different baseplates were placed. Baseplates had multiple magnet strips glued on, allowing baseplates to be firmly placed on the metal panel letting participants grasp blocks without pulling the baseplates and permitting researchers to quickly replace baseplates for each assembly in a matter of seconds. In addition, to provide consistency in the position of the baseplates the metal panel had markers that indicated the required position of the baseplates. Fig. 2 shows the back of a panel with magnetic strips.

## Sequence

To perform the assembly task participants performed the following sequence. Firstly, they read a visual instruction pattern from left to right and from top to bottom. Secondly, using only their dominant hand, they selected and picked-up the blocks required for the assembly from a resource area. Thirdly, they placed these blocks on top of the blocks of the assembly area. The three areas are separated in three different Lego Duplo® panels and contained Lego Duplo® blocks with distinct colors, white blocks for the visual instructions (1), green blocks for the assembly area (2) and blue blocks for the resource area (3). A representation of the experimental layout containing these areas is shown in the first figure in the original manuscript [3].

The visual instruction area contained a random arrangement of 12 blocks (6 square and 6 rectangular), the rectangular blocks had vertical and horizontal orientations. The visual instruction area provided a space between blocks, so participants could easily distinguish the required assembly block and its orientation Fig. 3.

The resource area contained the required assembly blocks in the required orientations, and between 5 and 7 distractor blocks. The distractor blocks had similar shapes and color hues as the required assembly blocks but did not meet the exact block shape or color hue of the required blocks. For example, some distractors were longer (2 × 6) or shorter (1 × 2) than the (2 × 4) required rectangles, or a square block had a transparent hue or a different coupling shape. In addition, the number of blocks in the resource area and their position were cycled through ten patterns Fig. 4. The rationale is that participants would need to seek for the required blocks while getting visually distracted.

**Fig. 5:**
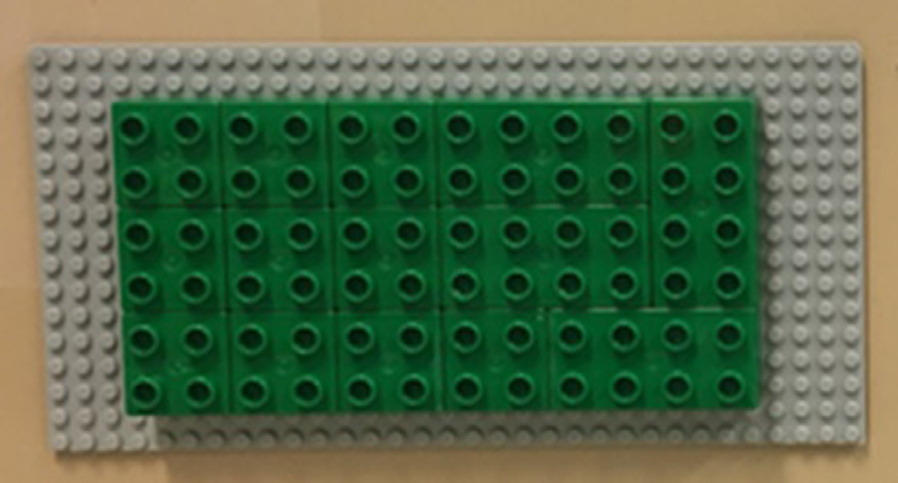
Assembly Area.

In addition, we learned that block assembly was performed easier over blocks rather than baseplates. Therefore, the assembly area Fig. 5 had glued green blocks on the baseplate. This prevented green blocks from falling as the blue blocks were assembled on top of them. Moreover, green blocks were useful for differentiating the different assembly areas by providing a clear visual cue between assembled and unassembled blocks.

## Assembly posture

**Fig. 6:**
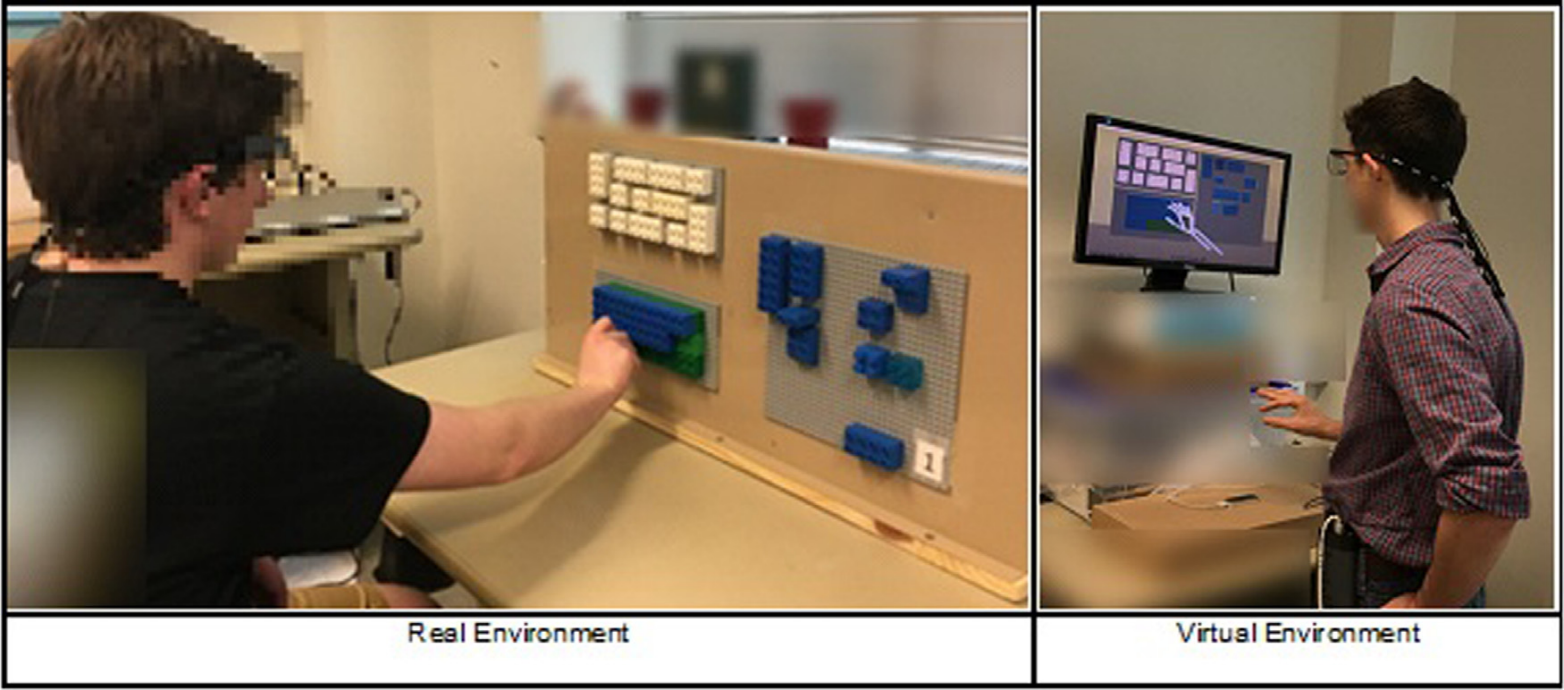
Assembly posture for real and virtual environments.

Under the real environment, participants performed the assembly task seated with the different panels placed on top of a steel panel as shown in Fig. 6 [3]. On the other hand, the virtual assembly required participants to stand as shown in Fig. 6 [3]. After completing an assembly cycle in the real environment participants were instructed to rotate their chair to the left side to allow experimenters to replace the baseplates for the resource and assembly areas. This rotation also prevented participants to observe and memorize the visual instruction pattern (Fig. 3) while they were not performing the assembly task.

The virtual environment was displayed on a 24″ computer monitor at a resolution of 1080p (1920 × 1080 pixels, 16:9 aspect ratio). We chose to project the virtual environment using a computer monitor because the eye tracking glasses did not fit inside of a helmet-mounted display.

As described, the virtual assembly required participants to stand. Having a standing position prevented participants’ visual confusion as their real hand was placed above the LEAP® motion controller and below their eyesight preventing a confusion from observing their virtual and real hand simultaneously. Therefore, this setting allowed participants to only observe their virtual hand on the computer screen. Participants stood at an approximate distance of 60 cm from the computer screen. Participants stand on a marking on the floor to maintain consistency. The computer monitor's height was adjusted to each participants’ eye height. In addition, after completing an assembly cycle in the virtual environment participants had a ten second pause were a scenic image was displayed. This delay had two purposes, first it allowed participants to lower their hand and to rest from the assembly position, and second it provided observation consistency by preventing participants to observe and memorize the visual instruction pattern (Fig. 1) while not performing the experiment.

## Virtual system safeguards

In addition, to posture differences, the virtual assembly process included additional visual and sound cues to support the virtual assembly. Sound cues included a “click-like” cue to indicate when the user acquired control of a block and an “assembly” cue to indicate when a block had been placed. The visual cues delivered a change in block color from blue to red, if the depth location of the assembly block was either too close or too far from the required assembly area. A change from blue to grey if the block was positioned at the correct assembly position. Having a color change greatly helped participants to determine their depth perception in the virtual environment. Moreover, to provide a close visual perception between environments the scale of the virtual representation of the assembly blocks was set at a 1:1 scale.

The virtual environment included several safeguards to warrant that the assembly would be done properly. First, blocks were programmed to be placed in the required sequence, and participants could not assemble a block if the previous required block in the sequence was not properly placed. Second, an assembled block could not be re-activated after being properly placed. Third, if a block was selected and for some reason the participant lost control over the block, participants were able to regain control of the block at its current location. However, if they did not regain control of the block within the following 2 s the block would go back to its original location.

## Computer and eye-tracking hardware and eye-tracking mapping

Our experimental setting used two computers with the same characteristics, an Intel Core i7–6500 U processor @ 2.50 GHz with an installed physical memory (RAM) of 16.0 GB and a dedicated video card NVIDIA GeForce 940 M with a 2GB video memory size and core clock speed of 1072 MHz/1176 Mhz. One of the computers was used to control the virtual environment and the other computer was used to control the eye-tracking hardware. The virtual environment was developed using Unity 5.4.2f2 (64bit) and was manipulated using a LEAP® Motion controller. The LEAP® controller version was 3.1.3 + 41,910. The LEAP® controller has a field of view of 150 × 120° and uses two 640 × 240 infrared cameras with a framerate of 120 frames per second.

The eye-tracking movements were captured using a Tobii Pro-Glasses 2® eye tracker. The Tobii Pro-Glasses 2® has four eye-tracking sensors with a sampling rate of 100 Hz with a field of view of 90°. The Tobii Pro-Glasses 2® are connected to a recording unit and a computer, and the recorded information was later mapped using the Tobii Analyzer® Software version 1.36.1430 (x64). The Tobii Analyzer® Software was run in one of the two computers previously described using an AOC computer monitor model 270LM00029 to run the VE and the Tobii Analyzer® Software.

The Monitor display is LED-backlit LCD monitor with an aspect ratio 16:9 and a resolution of Full HD (1080p) 1920 × 1080 at 60 Hz with the following dimensions (W x D x H) 24.5 in x 5.4 in x 18.1 in.

Obtaining results for the experiment required some preparation time as eye-tracking information was mapped from three snapshots into the previously described five regions of interest (ROIs) per assembly cycle for all participants. In addition, the analyzer took about 10–15 min for mapping and processing per participant per environment. Moreover, the eye-tracking report required additional post-processing to include the information from all cycles and participants. Therefore, this was a time-consuming process that required several days.

## Region of interest (ROI) coding

**Fig. 7:**
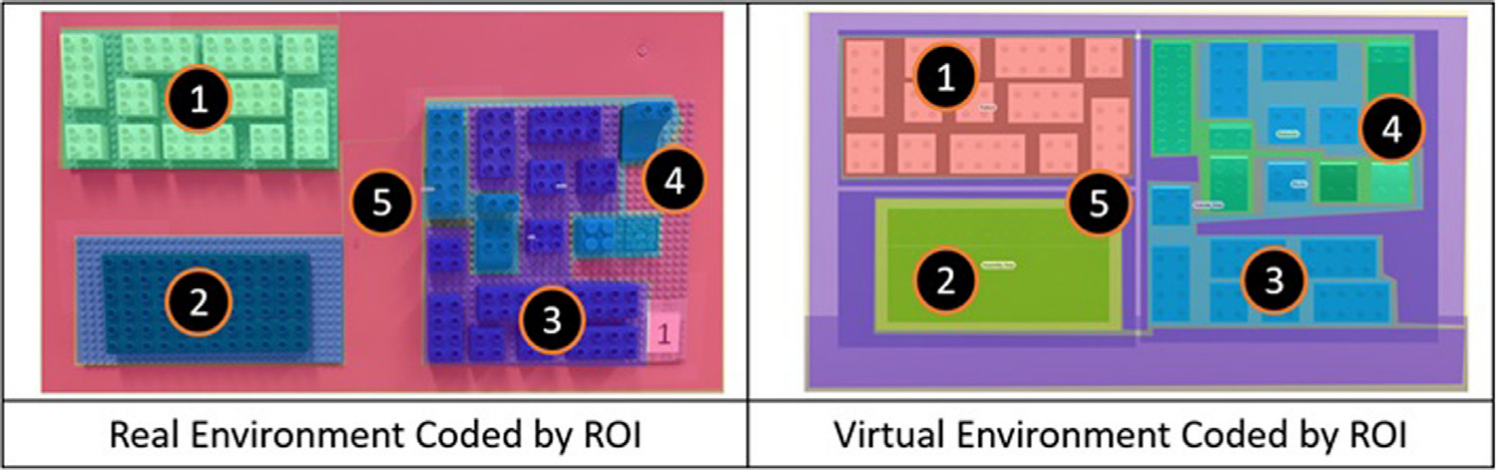
Coded regions of interest for real and virtual environments [6].

Given the nature of the eye-tracking research we coded five regions of interest (ROIs) in the assembly layout as seen in the Fig. 7 [3].

Region one is the visual instruction pattern shown in Fig. 3. Region two is the assembly area shown in Fig. 5. Regions three (assembly blocks) and four (distractor blocks), are in one of the ten different resource areas Fig. 4. Finally, region five corresponds to the outside area, an area which connects all other areas.

## Results

Since participants either started in the Real Environment (RE) first or the virtual environment first (VE), we compared assembly times per cycle for four conditions: RE with no training, RE with VE training, VE with no training, and VE with RE training. The training for the real environment was received by performing the assembly task in the virtual environment and the training for the virtual environment as received by performing the assembly task in the real environment.

We ran a set of planned paired two-tailed *t*-test comparisons only examining assembly duration differences for between VE and RE for participants with no previous training, At cycle 1, RE participants were statistically faster than VE participants, t(14) = −7.12, *p* < 1e-4, *d* = 2.60. Similarly, for the 10th assembly, RE participants were also significantly faster than VE t(14) = −9.31, *p* < 1e-6, *d* = 6.40 [6].

**Fig. 8:**
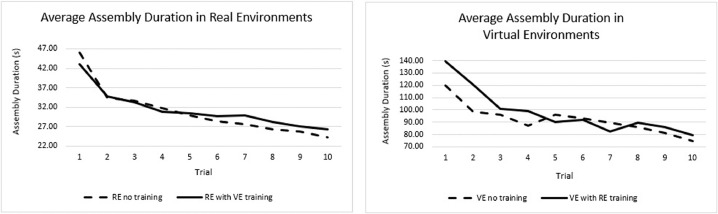
Average assembly duration per environment with and without previous training [6].

Moreover, Fig. 8 shows the average assembly duration per environment with and without previous training and the effect of practice.

**Fig. 9:**
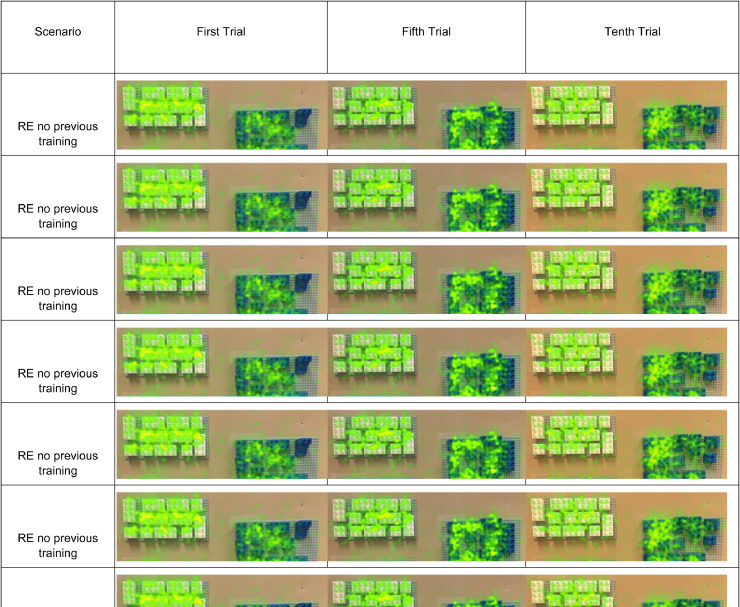
Heatmaps for the first, fifth and tenth assembly cycle for both environments, with and without previous training [3].

Similarly Fig. 9 shows the heatmaps for the first, fifth and tenth assembly cycle for both environments, with and without previous training. The areas with high proportion of eye-fixations are marked with red, while the medium proportion areas are in yellow and the low proportion areas are in green. The heatmap shows that the assembly area and the blocks have a higher incidence of eye-fixations in comparison to other ROIs. More information about these results is shown in the original manuscript [3].

## Discussion and future work

This method allows the identification of the key visual areas for both environments, and how training affects the observation of these areas. Moreover, it shows the observation similarities and differences between environments. For example, as described in the original manuscript “our results indicate that both environments share the same relevant ROIs (assembly area and blocks), but the observation proportion for key visual areas is higher for VEs” [3]. Showing how the interaction with VE affect the way we observe a VE.

In addition, the results from this method could be improved by incorporating analysis of saccades, pupil diameter, scan paths, and subjective multidimensional assessment tools to assess participants’ perceived workload such as the application of the NASA TLX, as described by NASA the NASA TLX “is a subjective workload assessment tool to allow users to perform subjective workload assessments on operator(s) working with various human-machine interface systems” [4].

**Supplementary material and/or Additional information:** [OPTIONAL. We also give you the option to submit both supplementary material and additional information. Supplementary material relates directly to the work that you have submitted and can include extensive excel tables, raw data etc. We would also encourage you to include failed methods or describe adjustments to your methods that did not work. Additional information can include anything else that is not directly related to your method, e.g. more general background information, useful links etc. Introduction is not a section included in the MethodsX format. This information could be moved to the end under Additional Information.

## Appendix Supplementary materials

Image, application 1

Image, application 2

Image, application 3
